# The Clinical Society

**Published:** 1898-12-24

**Authors:** 


### THE CLINICAL SOCIETY.

At the last meeting of the Clinical Society Mr. Campbell Williams described a curious case which seemed to show that the virus of syphilis could remain active in the dried state for over two months. The vehicle by which the infection was conveyed was in this case the mouthpiece of a set of bagpipes which had been played upon by a syphilitic subject and then put away for two months, when the owner used them and afterwards developed a hard sore on the tongue, which was followed by the usual secondary sequelæ. The chancre appeared either 20 or 30 days after the presumed infection from the bagpipes. Mr. Clement Lucas said that there was no more improbability in the preservation of dried syphilitic virus than of dry vaccine on the old ivory points. The principal interest of the evening meeting, however, lay in the series of papers dealing with the pancreas and its diseases. Mr. Pearce Gould related a case of pancreatic calculi, one of which obstructed the bile duct. They were removed by two operations, and the biliary obstruction was relieved, but the patient died. Mr. A. D. Fripp related a case of acute hæmorrhagic pancreatitis, with extensive fat necrosis, which also died; and Dr. Newton Pitt and Mr. Jacobson gave a paper on five cases of acute pancreatitis, four of which ended fatally.

